# Supplementary material for: Respiratory symptoms and pulmonary function in paint industry workers exposed to volatile organic compounds: A systematic review and meta-analysis
Source: PLoS One. 2024 Dec 26;19(12):e0315464. doi: 10.1371/journal.pone.0315464 (PMC11671006; doi:10.1371/journal.pone.0315464)
Supplement: S2 Table — (DOCX) [file pone.0315464.s003.docx]

**Respiratory symptoms and Pulmonary function in paint industry workers: A Systematic Review and Meta-Analysis**

| **Supplementary 2: Modified Newcastle Ottawa Scale quality Assessment** | | | | | | | | | |
| --- | --- | --- | --- | --- | --- | --- | --- | --- | --- |
| [**SI.NO**](http://si.no/) | **Cross sectional Studies** | **Selection** | | | | **Comparability** | **Outcome** | | **Quality** |
|  |  | **Representativeness of the sample** | **Sample size** | **Non respondents** | **Ascertainment of exposure** | **Based on study design & analysis** | **Assessment of outcome** | **Statistical test** |  |
|  | (Schwartz and Baker 1988) | ***** |  | ***** | ***** | ***** | ***** | ***** | 6 |
|  | (Alexandersson and Hedenstierna 1988) | ***** |  | ***** | ****** | ***** | ***** | ***** | 7 |
|  | (Eifan et al. 2005) | ***** | ***** | ***** | ***** | ***** | ***** | ***** | 7 |
|  | (Hammond et al. 2005) | ***** | ***** | ***** | ****** | ***** | ***** | ***** | 8 |
|  | (Kaukiainen et al. 2005) | ***** |  | ***** | ***** | ***** | ***** | ***** | 6 |
|  | (Ould-Kadi et al. 2007) | ***** |  | ***** | ***** | ***** | ***** | ***** | 6 |
|  | (Kaukiainen et al. 2008) | ***** |  |  | ***** | ***** | ***** | ***** | 5 |
|  | (El Mahdy and Radwan 2009) | ***** |  |  | ****** | ****** | ***** | ***** | 7 |
|  | (Metwally et al. 2012) | ***** |  |  | ***** | ***** | ***** | ***** | 5 |
|  | (Numan 2012) | ***** | ***** |  |  | ***** | ***** | ***** | 5 |
|  | (El-Gharabawy et al. 2013) | ***** |  |  | ***** | ***** | ***** | ***** | 5 |
|  | (Mandal and Majumdar 2013) | ***** |  |  | ****** |  | ***** | ***** | 5 |
|  | (Hakim et al. 2014) | ***** | ***** | ***** | ***** | ***** | ***** | ***** | 7 |
|  | (Aribo and Antai 2014) | ***** |  | ***** | ***** | ***** | ***** | ***** | 6 |
|  | (Ojo et al. 2017) | ***** |  |  | ****** | ****** | ***** | ***** | 7 |
|  | (Khode and Komal 2017) | ***** |  |  | ***** | ****** | ***** | ***** | 6 |
|  | (Hagras et al.2017) | ***** |  |  |  | ****** | ***** | ***** | 5 |
|  | (Onesmo and Rongo 2018) | ***** |  | ***** |  | ***** | ***** | ***** | 5 |
|  | (Hwang et al. 2018) | ***** |  | ***** | ****** | ***** | ***** | ***** | 7 |
|  | (Saraei et al. 2019) | ***** |  |  |  | ***** | ***** | ***** | 5 |
|  | (Jabbar and Mohammed 2020) | ***** |  |  | ***** | ****** | ***** | ***** | 6 |
|  | (Ahmad and Balkhyour 2020) | ***** | ***** |  | ***** | ***** | ***** | ***** | 6 |
|  | (Ojo et al. 2020) | ***** |  |  | ****** | ****** | ***** | ***** | 7 |

**Newcastle-Ottawa Scale adapted for cross-sectional studies**

**Selection:**

1. Representativeness of the sample:
   1. Truly representative of the average in the target population. * (all subjects or random sampling)
   2. Somewhat representative of the average in the target group. * (non-random sampling)
   3. Selected group of users/convenience sample.
   4. No description of the derivation of the included subjects.
2. Sample size:
   1. Justified and satisfactory (including sample size calculation). *
   2. Not justified.
   3. No information provided
3. Non-respondents:
   1. Proportion of target sample recruited attains pre-specified target or basic summary of non-respondent characteristics in sampling frame recorded. *
   2. Unsatisfactory recruitment rate, no summary data on non-respondents.
   3. No information provided
4. Ascertainment of the exposure (risk factor):
   1. Vaccine records/vaccine registry/clinic registers/hospital records only. **
   2. Parental or personal recall and vaccine/hospital records. *
   3. Parental/personal recall only.

**Comparability:** (Maximum 2 stars)

1. Comparability of subjects in different outcome groups on the basis of design or analysis. Confounding factors controlled.
   1. Data/ results adjusted for relevant predictors/risk factors/confounders e.g. age, sex, time since vaccination, etc. **
   2. Data/results not adjusted for all relevant confounders/risk factors/information not provided.

**Outcome:**

1. Assessment of outcome:
   1. Independent blind assessment using objective validated laboratory methods. **
   2. Unblinded assessment using objective validated laboratory methods. **
   3. Used non-standard or non-validated laboratory methods with gold standard. *
   4. No description/non-standard laboratory methods used.
2. Statistical test:
   1. Statistical test used to analyse the data clearly described, appropriate and measures of association presented including confidence intervals and probability level (p value). *
   2. Statistical test not appropriate, not described or incomplete.

Cross-sectional Studies:

Very Good Studies: 9-10 points

Good Studies: 7-8 points

Satisfactory Studies: 5-6 points

Unsatisfactory Studies: 0 to 4 points

This scale has been adapted from the Newcastle-Ottawa Quality Assessment Scale for cohort studies to provide quality assessment of cross sectional studies^[[1]](#footnote-1)^

1. Herzog R, et al. Is Healthcare Workers’ Intention to Vaccinate Related to their Knowledge, Beliefs and Attitudes? A Systematic Review. *BMC Public Health* 2013 **13**:154 [↑](#footnote-ref-1)
