# Supplementary material for: Respiratory symptoms and pulmonary function in paint industry workers exposed to volatile organic compounds: A systematic review and meta-analysis
Source: PLoS One. 2024 Dec 26;19(12):e0315464. doi: 10.1371/journal.pone.0315464 (PMC11671006; doi:10.1371/journal.pone.0315464)
Supplement: Attachment — Submitted filename: Response to reviewers (2).docx [file pone.0315464.s004.docx]

**PONE-D-24-08732**

**Respiratory symptoms and Pulmonary function in paint industry workers: A Systematic Review and Meta-Analysis**

**Dear Editor in-chief,**

We appreciate your consideration of our manuscript for the peer review procedure. The valuable feedback and suggestions provided by the reviewers to refine our current manuscript have been valued sincerely. To the best of our ability, we have responded to each comment with supplementary information and clarifications, while also adhering to the PlosONE guidelines for review articles.

**Response to reviewers**

Thank you for your valuable insights and constructive feedback regarding our systematic review and Meta-analysis.

**Reviewer #1:**

**Query #1:This is an original systematic review of a relevant topic. In general the selection of articles seems to have been done rigorously leading to a comprehensive inclusion of relevant studies published in English. Nevertheless, the non-inclusion of articles on the Ardystil disease caused by spray painting among textile workers is conspicuous (and should be justified).**

**Response:** Thank you for your thoughtful comment. Our current research is primarily focused on investigating the respiratory health effects of Volatile Organic Compounds (VOCs). Nevertheless, Ardystil syndrome is predominantly associated with the inhalation of aerosolized polyacrylic resins, (which is not a VOC) such as Acramin, which are frequently used in textile printing. This condition is mainly linked to three primary polycationic paint components: Acramin FWR (a polyurea), Acramin FWN (a polyamide-amine), and Acrafix FHN (a polyamine). Of these, Acramin FWR has been implicated in the development of interstitial lung disease among workers using the airbrushing technique.

Hence, inclusion of such studies would fall outside the specific scope of our current research. This is the reason why they were not included in this systematic review. However, we recognize the importance of these studies and plan to undertake a separate systematic review in the near future to explore the toxicological effects of other paint components, including Acramin and similar substances.

**Query #2:** **I have the following concerns regarding methodology:** **Please, indicate the start date for the inclusion of articles (line 84, line 169).**

**Response:** Thank you for your feedback regarding the methodology. In accordance with your suggestion, we have specified the start date as "from inception". This designation ensures that our review includes all relevant research articles published from their inception up to August 2024.

**Pages: 5,12	Lines: 86, 171**

**Query #3: line 105 (iii) confusing formulation: "inclusive of ..." to be replaced by "covering" or "dealing with ..."? what do you mean by "or organic solvents" here (as opposed to VOCs)?**

**Response:** Thank you for your feedback. We have revised the formulation as per your suggestion, replacing "inclusive of ..." with "covering" to enhance clarity. Additionally, we have removed the phrase "present in paints or organic solvents" to avoid any ambiguity.

**Page:6 Line: 106**

**Query #4:** **lines 174-175: Iraq is not an African country! Baghdad and Tehran are not countries but country capitals. These troublesome errors have an impact on the meta-analysis by region!**

**Response:** Thank you for pointing out these errors. We apologize for the oversight. We have corrected the inaccuracies regarding Iraq, Baghdad, and Tehran, ensuring accurate representation in both the methodology and results sections. Your suggestion has been invaluable in improving the integrity of our meta-analysis by region.

**Page: 12 Lines: 175-179, Table:2,6**

**Query #5:** **table 4: values of FEV1 and FVC can be/are reported as percentages of predicted values (note implausible values for study #16)) or as absolute values (with corrections for sex, age and height?);**

**Response:** Thank you for your observation regarding Table 4. We have addressed the issue, specifically for study #16, by reporting FEV1 and FVC values as percentages of predicted values. This adjustment ensures accuracy and consistency in our presentation of the data.

**Pages: 16 Line: 239**

**Query #6: FEV1/FVC ratios are reported as percentages, most being plausibly around 80% (FEV1/FVCx100) but studies #8 and 14 have values above 90%, presumably because they were expressed as percent of a predicted ratio (not a good option!). HOW WERE THESE DIFFERENCES IN THE EXPRESSION OF SPIROMETRIC PARAMETERS TAKEN INTO ACCOUNT FOR THE QUANTITATIVE ANALYSES ? Even when using SMDs, this issue is crucial for the general credibility of the analyses and conclusions.**

**Response:** As correctly stated, studies #8 and #14 presented the FEV1/FVC ratio values as predicted percentages, while the remaining studies reported them as absolute values. To maintain consistency across the studies, we standardized the mean differences of the reported FEV1/FVC ratios in our quantitative analysis. This approach allowed us to maintain a high level of consistency in both the calculation and interpretation of spirometry parameters across the entire dataset. We would like to highlight that we have computed the standardized mean difference of the exposed and unexposed groups, which were represented as either absolute values or predicted values. It is of utmost importance to ensure that the conclusion derived from our meta-analysis accurately represents the combined evidence from the included studies. We would like to extend our sincere apologies for the oversight regarding the absence of the " * " marking in study #8, which might have caused confusion.

In addition, we would like to cite an article that includes calculations of mean difference FEV1/FVC ratios expressed as a percentage of predicted values.

Jing Z, Wang X, Zhang P, Huang J, Jia Y, Zhang J, Wu H, Sun X. Effects of physical activity on lung function and quality of life in asthmatic children: An updated systematic review and meta-analysis. Frontiers in Pediatrics. 2023 Feb 8;11:1074429.

**Query #7:** **Tables: please include the corresponding reference numbers (in the list of references) in all tables and figures.**

**Response:** As per the suggestion, we have included the corresponding reference numbers (in the list of references) in all tables and figures.

**Query #8:** **Table 2: in the outcome column, the text can/should be much more concise (avoid unnecessary words "symptoms like ...") and make a clearer distinction between symptoms and PFTs (perhaps using separate columns).**

**Response:** Thank you for your feedback regarding Table 2. We have updated the table to separate Respiratory symptoms and Pulmonary Function Tests (PFTs) into distinct columns. Additionally, we have revised the text in the outcome column to ensure conciseness, eliminating unnecessary wording such as "symptoms like ...". These changes enhance the clarity and organization of the data presented in the table.

**Query #9: Tables 3, 5 and 6: add good legends**

We have added good legends in the above tables based on your suggestion.

**Query #10: Table 4: see comments made above about expression of parameters; legend: ** values in liters (not litres/minute), last phrases of legend make no sense**

**Response:** The necessary modifications have been made in Table.4.

**Query #11: Figure 2, 3, 4, 5: please harmonize the styles across figures, add suitable explanatory legends, specify units as needed, check and harmonize how study authors are identified**

**Response:** We have harmonized the style across the figures, added suitable explanatory legends, specified the units as needed and harmonized the style across study authors as well.

**Query #12:** **Discussion: the (speculative) mechanistic explanations (lines 323-330) may be deleted.**

**Response:** We have removed the speculative mechanistic explanations from the discussion section as recommended.

**Query #13: line 392: gaps were not really identified: suggestions are: why, unusually, so few studies from industrially developed countries? what about prevalence of clinical pulmonary disease (asthma or COPD), what about pulmonary fibrosis? What is the impact in terms of overall morbidity and mortality (proportion of painters in the population)? What is the public health impact of painting on respiratory health compared to other occupational exposures (mining, construction,….)? What about different types of paints (solvent-based vs water-based paints) and application modes (spraying vs brush)?**

**Response:** We have added the identified gaps and made the necessary changes in the lines starting from 392 in accordance with the feedback.

**Page: 23,24	Lines:396 -407**

**Query #14: Language: generally OK, but some editing is needed (pulmonary function is generally singular; "the effect reduced", ...).**

**Response:** We have incorporated the reviewer’s suggestions and made the necessary changes in the entire manuscript.

**Reviewer #2:** The present study is a systematic review and meta-analysis to determine pulmonary function and symptoms in paint industry workers. The review of the literature, the qualitative evaluation of the articles and the calculation of the pooled index were done using an appropriate scientific method. The presentation of results is appropriate and in the discussion section, interpretation has made based on the obtained results.

**Response:** Thank you for your positive feedback and appreciation of our study. We are pleased that you found our systematic review and meta-analysis methodology appropriate and the presentation of results satisfactory. Your comments encourage us to continue our efforts in contributing to the field of occupational health research.

**Reviewer #3:**

Thank you for your valuable insights and constructive feedback regarding our systematic review and Meta analysis.

Major issues:
**Query #1:** **I would suggest to remove any speculation on the obstructive disorder, including those in the conclusions, because the review did not analyze the obstructive disorder as such, but rather a SMD in FEV1/FVC in the exposed vs unexposed groups. Even despite some identified difference (through SMD), obstruction has clear and distinct diagnostic criterion which is FEV1/FVC below LLN or when LLN is not available, below 70%. Therefore, obstructive defect can only be treated a binary variable. In other words, no such variable was analyzed in the review, and only the difference between the groups in FEV1/FVC should be discussed.**

Response: Thank you for your insightful perspective regarding the reporting of obstructive disorder in our review. We acknowledge that our study focused on the standardized mean difference (SMD) in FEV1/FVC between exposed and unexposed groups, rather than analyzing obstructive disorder as defined by specific diagnostic criteria. In response to your suggestion, we have removed or replaced such phrases and made the necessary adjustments accordingly.

**Query #2:** **Albeit some hint on the limitations related to no adjustment for potential confounders is made in the Discussion, this still remains a serious issue. Given that there is an array of confounders for both lung function and symptoms, including sex, regular exercise (please see** [**https://pubmed.ncbi.nlm.nih.gov/31920299/**](https://pubmed.ncbi.nlm.nih.gov/31920299/)**) and other exist, the discussion on that should be extended.**

**Response:** We appreciate your insightful comments regarding the limitations related to potential confounders in our study. The odds ratios mentioned in the study are the crude odds ratios calculated by the author. Unfortunately, the lack of data prevented us from taking into account potential confounders, such as age, and effect modifiers, such as exercise, in our analysis. In addition, we have made appropriate modifications in our discussion.

**Pages: 23, 24	Lines:387-389, 414 -416**

**Query #3:Please speculated over no males in the analysis and even more important – no adjustment in the effect for confounders.**

**Response:** We appreciate your query, it is important to note that the studies included in the analysis primarily concentrated on male populations, with only two exceptions that specifically investigated respiratory symptoms.

**Pages: 22,15, 22	Lines: 369 -371,235,369,**

**Query #4:** **This comment also extends to the comment for crude effects calculation below. When you summarized the overall effect in the conclusions (including the abstract), please state that all effects were crude, and the effects of confounders was omitted.**

**Response:** Thank you for your suggestion. We have incorporated your recommendation by explicitly stating in the abstract, results, discussion, and conclusion sections that the odds ratios reported for respiratory symptoms are crude estimates, as confounding factors were not adjusted for in our analysis.

**Pages: 13,14,18,24 Lines:45, 202, 203, 208, 209, 215, 223, 286, 287, 292, 293, 294, 387-389, 414 -416.**

**Query #5: Despite some discussion on very high heterogeneity, a serious concern remains for almost all polled analysis in this report, including those where I2 reached 99%. This is an extreme heterogeneity, and apparently more discussion is needed on the sources of heterogeneity. I suppose these could be surprisingly low samples in selected studies, introducing very poor precision. This of other sources and discuss this extreme heterogeneity in more detail.**

**Response:** As you rightly stated one of the probable reasons for heterogeneity has been discussed in the discussion section.

**Page: 22	Lines:372 - 376**

**Query #6: Overall, conclusions in this review are constructed on cross-sectional studies, assuming poor evidence. Limitations of this design are well-known, but no temporality and other limitations should be louder pronounced in the results and even more in the discussion and conclusions. This is a very important finding.**

**Response:** As per your suggestion, we have documented about the limitations of the study group in the results,discussion and in conclusion sections.

**Pages: 23,24 Lines:382-387,413-414**

**Query #7:** **De-abbreviate VOC at first use in the abstract and in the main text. Same relates to PFT in line 121. Furthermore, when you have already abbreviated VOC in the beginning, use only VOC later on (line 229), and check all over the manuscript.**

**Response:** The abbreviation for VOCs, PFT have been expanded upon its first use, both in the abstract and in the main text. Additionally, we have incorporated the abbreviated form throughout the manuscript based on your valuable suggestions.

**Pages:3,4,6 Lines:36,43,59,121**

**Query #8:** **Use a uniform term across the manuscript. At present, you use “pulmonary function”, “respiratory function”. Moreover, the terms “pulmonary functions” as plural is usually not used; instead, use “pulmonary function” if you chose this out of two (“pulmonary function” vs. “respiratory function”).**

**Response:** As per your recommendation, respiratory function has been uniformly changed to pulmonary function.

**Query #9:** **Abstract: The aim in the abstract only mentions pulmonary function. But the title and results all over the manuscript also mention symptoms.**

**Response:** We have included the respiratory symptoms under “Aim” in the abstract which was missed inadvertently earlier.

**Query #10:** **Please fix this discrepancy. Results: delete “of the lungs” when you report SMD for FEV1 and FEV1/FVC.**

**Response:** We have deleted the phrase "of the lungs" as per your request.

**Query #11: Conclusions: these conclusions are poorly focused. Please make them succinct.**

As per your suggestion, we have made the necessary changes in conclusion

**Page: 24	Lines: 409 -425**

**Query #12: Introduction: Many statements in the Introduction do not have references, starting with the very first sentence. Is you narrate the exposure has detrimental effects, then present references that confirm the association.**

**Response:** We have added the references in the “Introduction” section as suggested.

**Page: 24	Lines: 409 -425**

**Query #13: In lines 67-68 you refer to several studies, but cite only one. Same relates to lines 72-75.**

**Response:** We have cited as per your suggestion. Apologies for missing the same earlier.

**Query #14: Why was the age limit 15-60 set as a selection criterion?**

The age limit of 15-60 years was chosen as the 15 to 60 age range encompasses the majority of the working-age population, during which lung function tends to stabilize and remain relatively consistent. This age bracket is considered ideal for obtaining reliable and comparable measurements of lung function. Below the age of 15, lung function vary significantly due to growth spurts and developmental changes. By age 15, most individuals reach a stage of physiological maturity where their lung function closely resembles that of adults. Age beyond 60 years can lead to anatomical and functional changes in respiratory functioning, which may introduce variability in study results. Therefore, to ensure consistency and reliability in our study's measurements and to minimize potential confounding factors related to age-related changes in lung function, we have established an age limit of 15 to 60 years for our participant selection criteria. We can include these reasons in our manuscript to provide further clarity if recommended by the reviewer.

**Reference:**

1. Quanjer PH, Stanojevic S, Cole TJ, Baur X, Hall GL, Culver BH, Enright PL, Hankinson JL, Ip MS, Zheng J, Stocks J; ERS Global Lung Function Initiative. Multi-ethnic reference values for spirometry for the 3-95-yr age range: the global lung function 2012 equations. Eur Respir J. 2012 Dec;40(6):1324-43. doi: 10.1183/09031936.00080312. Epub 2012 Jun 27. PMID: 22743675; PMCID: PMC3786581.

2. Townsend, M.C. (2011). "ACOEM Position Statement: Spirometry in the Occupational Setting." Journal of Occupational and Environmental Medicine, 53(5), 569-584. https://doi.org/10.1164/rccm.201402-0337ST

**Query #15: Line 143: what range? Interquartile range? Moreover, what dictated the choice between reporting means and medians? Normality? Then state that.**

**Response**: Thank you for your insightful inquiry. The range in line 143 (previous version) represents the minimum to maximum mean values. Based on the suggestion of our statistician, we have computed the combined mean and standard deviation for all descriptive study variables, such as age and years of experience for both PFT and respiratory symptoms.In addition, determining normalcy may pose a challenge due to the limited availability of raw data.

**Query #16:** **Line 144: who calculated ORs? Did the authors of this review did that? If so, how then 95% CIs were computed? Furthermore, if the authors of this review did the computation but not originally reported in the source articles, this assumes that all computed ORs were crude, and no adjusted for confounders was possible. If that is the case, discuss that in the limitations. This should also be clearly stated in the footer to Table 3.**

**Response:** Yes, the odds ratios with the 95% confidence interval reported in our review were calculated by the authors. We derived these calculations based on data extracted from the included studies, following standard methods for meta-analysis synthesis. The 95% confidence intervals for the calculated odds ratios were determined using the approach outlined in Altman DG's 'Practical Statistics for Medical Research' (1991).

It is important to note that all odds ratios reported in this review are crude estimates. We did not have access to individual participant data from the primary studies, which prevented us from calculating adjusted odds ratios that would account for potential confounders. Therefore, our findings reflect associations based on unadjusted odds ratios across the included studies.

In the limitations section, we have acknowledged that the odds ratios reported were crude and were not adjusted for confounders due to data limitation. Additionally, as per your suggestion, we have included a footer in Table 3 stating that all odds ratios are represented as crude ratios which reflects that they were not adjusted for potential confounders.

**Page: 13, 18, 23 Lines:195, 297, 387 - 389**

**Query #17: Line 145: the rationale to report SMD is not provided. Please clearly state that some studies reported actual FEV1 or FVC or the ratio, but some of them reported %predicted values. Therefore, direct comparison was not possible, and SMD calculation was needed.**

**Response:** As per your suggestion, we have given the rationale for calculating the SMD in the data synthesis and analysis section of the methodology.

**Page: 8 Lines: 145 -148**

**Query #18:** **Results: Lines 168-180: state that none of included studies applied case-control or cohort design. This is a very important finding, assuming the despite quite high scores, overall the quality of evidence is quite low, since cross-sectional design has serious limitations.**

**Response:** We have carefully considered your input and have mentioned that the selected studies were cross-sectional in design. Further, we have comprehensively addressed this limitation in the discussion part of the paper.

**Page: 24 Lines: 383, 384, 382 -387**

**Query #19: In addition, summarize how many studies used questionnaires for exposure assessment as opposed to measured exposure. Any studies using JEMs?**

**Response:** Although the information on the source of exposure assessment was provided in Table.2, we have summarised this information under the “ Study Characteristics” section of methodology as per your suggestion.

**Page: 12 Lines:182 -184**

**Query #20: Results: how would you explain that subgroup analysis of FEV1 and FVC with regard to smoking yielding opposite results? Is the reason very few eligible studies in the FVC analysis?**

**Response:** As rightly pointed out by you, there are only two studies that categorized people as smokers or non-smokers and had evaluated the FVC. Therefore the results may not be consistent. In addition, the overall estimate [SMD:-2.14 (-5.04,0.77)] and the subgroup estimates [SMD among smokers:-0.35(-0.53,-0.18), and SMD of Nonsmoker:-0.54(-0.76,-0.33)] of FVC did not yield significant results. Therefore, it is difficult to draw a consistent conclusion based on these findings. In order to provide readers with a more comprehensive understanding, we will indicate that FVC did not produce any significant results in the results section.

**Page: 20 Lines:314,315**

**Query #21: Discussion: Normally tables and figures are not cited in the discussion. Please remove that and leave only text.**

**Response:** Tables and figures cited in the discussion have been removed as suggested.

**Query #22: Language needs more effort. Please proofread the text. One of the problems is sequence of tenses. Carefully check grammar. In addition, some typos (line 346: wide) are also present.**

**Response:** Your suggestion and identification of the typo error are greatly appreciated.We have carefully reviewed the article and have made the necessary corrections to address any typos, grammatical errors, and language issues, in accordance with your suggestions.I would like to inform you that this manuscript received language editing from a professional service (Editage) before being submitted to Plos ONE. We would like to inform you that there may have been some typographical or grammatical errors that were overlooked. This happened as we made some modifications following the language editing process, just prior to submitting to Plos ONE.

**Query #23:** **Inclusion of only English studies is also a limitation and should be mentioned in the limitations.**

**Response:** We have incorporated the same in the limitation section (lines: 387 -389) as suggested.

**Page: 23	Lines: 389-391**
