# Supplementary material for: Respiratory symptoms and pulmonary function in paint industry workers exposed to volatile organic compounds: A systematic review and meta-analysis
Source: PLoS One. 2024 Dec 26;19(12):e0315464. doi: 10.1371/journal.pone.0315464 (PMC11671006; doi:10.1371/journal.pone.0315464)
Supplement: Attachment — Submitted filename: Response to reviewers_2.docx [file pone.0315464.s005.docx]

**PONE-D-24-08732**

**Respiratory symptoms and Pulmonary function in paint industry workers: A Systematic Review and Meta-Analysis**

**Dear Editor in-chief,**

We appreciate your consideration of our work for the peer review process. Thank you for providing us with valuable suggestions. We truly appreciate your input.

**#Comment 1**

**Please be aware of the suggestions provided by the Reviewers, especially those from the Reviewer 1 who still has some specific requests. I strongly recommend to make all the suggested changes to improve the manuscript.**

Thank you for taking the time to review our work. Your effort and input are greatly appreciated. We have carefully reviewed the suggestions given by reviewer 1 and have made the required revisions to the best of our ability. We have provided justifications and citations for your comments, particularly for queries 1, 6, 7, 10, 12 and 14 of reviewer 1. We have also thoroughly reviewed all the other comments by reviewer 1 to the best of our ability.

**Query #1:This is an original systematic review of a relevant topic. In general the selection of articles seems to have been done rigorously leading to a comprehensive inclusion of relevant studies published in English. Nevertheless, the non-inclusion of articles on the Ardystil disease caused by spray painting among textile workers is conspicuous (and should be justified).**

**Response:** Thank you for your thoughtful comment. Our current research is primarily focused on investigating the respiratory health effects of Volatile Organic Compounds (VOCs). Nevertheless, Ardystil syndrome is predominantly associated with the inhalation of aerosolized polyacrylic resins, (which is not a VOC) such as Acramin, which are frequently used in textile printing. This condition is mainly linked to three primary polycationic paint components: Acramin FWR (a polyurea), Acramin FWN (a polyamide-amine), and Acrafix FHN (a polyamine). Of these, Acramin FWR has been implicated in the development of interstitial lung disease among workers using the airbrushing technique.

Hence, **inclusion of such studies would fall** **outside the specific scope of our current systematic review**. **In addition, we have clearly mentioned in the exclusion criteria that studies dealing with major exposures other than VOCs were excluded.** However, we recognize the importance of these studies and plan to undertake a separate systematic review in the near future to explore the toxicological effects of other paint components, including Acramin and similar substances.

**Query #2:** **I have the following concerns regarding methodology:** **Please, indicate the start date for the inclusion of articles (line 84, line 169).**

**Response:** Thank you for your feedback regarding the methodology. In accordance with your suggestion, we have specified the start date as "from inception". This designation ensures that our review includes all relevant research articles published from their inception up to August 2024.

**Pages: 5, 12; Lines: 86, 171**

**Query #3: line 105 (iii) confusing formulation: "inclusive of ..." to be replaced by "covering" or "dealing with ..."? what do you mean by "or organic solvents" here (as opposed to VOCs)?**

**Response:** Thank you for your feedback. We have revised the formulation as per your suggestion, replacing "inclusive of ..." with "covering" to enhance clarity. Additionally, we have removed the phrase "or organic solvents" to avoid any ambiguity.

**Page: 6; Line: 106**

**Query #4:** **lines 174-175: Iraq is not an African country! Baghdad and Tehran are not countries but country capitals. These troublesome errors have an impact on the meta-analysis by region!**

**Response:** Thank you for pointing out these errors. We apologize for the oversight. We have corrected the inaccuracies regarding Iraq, Baghdad, and Tehran, ensuring accurate representation in both the methodology and results sections. Your suggestion has been invaluable in improving the integrity of our meta-analysis by region.

**Page: 12; Lines: 175-179, Table: 2, 6**

**Query #5:** **table 4: values of FEV1 and FVC can be/are reported as percentages of predicted values (note implausible values for study #16)) or as absolute values (with corrections for sex, age and height?)**

**Response:** Thank you for your observation regarding Table 4. We have made the necessary corrections in study #16, and reported FEV1 and FVC values as percentages of predicted values. This adjustment ensures accuracy and consistency in our presentation of the data.

**Page: 16; Table: 4**

**Query #6: FEV1/FVC ratios are reported as percentages, most being plausibly around 80% (FEV1/FVCx100) but studies #8 and 14 have values above 90%, presumably because they were expressed as percent of a predicted ratio (not a good option!).**

As correctly stated, studies #8 and #14 presented the FEV1/FVC ratio values as predicted percentages, while the remaining studies reported them as absolute values. We would like to extend our sincere apologies for the oversight regarding the absence of the " * " marking in study #8, which might have caused confusion.

**Page: 15, 16; Table: 4**

**Query #7: HOW WERE THESE DIFFERENCES IN THE EXPRESSION OF SPIROMETRIC PARAMETERS TAKEN INTO ACCOUNT FOR THE QUANTITATIVE ANALYSES? Even when using SMDs, this issue is crucial for the general credibility of the analyses and conclusions.**

**Response:** To maintain consistency across the studies, we performed standardized the mean differences (SMD) of the reported FEV1/FVC ratios in our quantitative analysis. i.e., we have computed the SMD of the exposed and unexposed groups, which were represented as either absolute or predicted values. It is important to note that the value of individual studies remains consistent regardless of the expressions of the spirometric parameters (absolute and predicted values). For better understanding, we performed a separate analysis in which we have categorized the studies into two groups: those studies that evaluated FEV1/FVC as an absolute value (Ref Fig 1) and those that evaluated predicted values (Ref Fig 2). It is worth noting that while comparing with the combined SMD (Ref Fig 3), the values of each study remains to be same. This demonstrates the ability to merge both expressions, resulting in improved clarity and analysis. It is of utmost importance to ensure that the conclusion derived from our meta-analysis accurately represents the combined evidence from the included studies.

We would like to cite few references regarding calculation of SMD’s for further clarification

- Andrade C. Mean difference, standardized mean difference (SMD), and their use in meta-analysis: as simple as it gets. The Journal of clinical psychiatry. 2020 Sep 22;81(5):11349.
- Gallardo‐Gómez D, Richardson R, Dwan K. Standardized mean differences in meta‐analysis: A tutorial. Cochrane Evidence Synthesis and Methods. 2024 Mar;2(3):e12047.

**Ref Fig 1(FEV1/FVC (absolute)	Ref Fig 2 (predicted %)**

**Ref Fig 3 (combined)**

**Query #8:** **Tables: please include the corresponding reference numbers (in the list of references) in all tables and figures.**

**Response:** As per the suggestion, we have included the corresponding reference numbers (in the list of references) in all tables and figures.

**Query #9:** **Table 2: in the outcome column, the text can/should be much more concise (avoid unnecessary words "symptoms like ...") and make a clearer distinction between symptoms and PFTs (perhaps using separate columns).**

**Response:** Thank you for your feedback regarding Table 2. We have updated the table to separate Respiratory symptoms and Pulmonary Function Tests (PFTs) into distinct columns. Additionally, we have revised the text in the outcome column to ensure conciseness, eliminating unnecessary wording such as "symptoms like ...". These changes enhance the clarity and organization of the data presented in the table.

**Query #10: Tables 3, 5 and 6: add good legends**

We reworked on it and added suitable legends to the above tables based on your valuable suggestion.

**Query #11: Table 4: see comments made above about expression of parameters; legend: ** values in liters (not litres/minute), last phrases of legend make no sense**

**Response:** The necessary modifications have been made in Table.4.

**Query #12: Figure 2, 3, 4, 5: please harmonize the styles across figures, add suitable explanatory legends, specify units as needed, check and harmonize how study authors are identified**

**Response:** We have harmonized the style across the figures, added suitable explanatory legends, specified the units as needed and harmonized the style across study authors as well.

**Query #13:** **Discussion: the (speculative) mechanistic explanations (lines 323-330) may be deleted.**

**Response:** We have removed the speculative mechanistic explanations mentioned in lines 323 – 330 from the discussion section as recommended.

**Query #14: line 392: gaps were not really identified: suggestions are: why, unusually, so few studies from industrially developed countries? what about prevalence of clinical pulmonary disease (asthma or COPD), what about pulmonary fibrosis? What is the impact in terms of overall morbidity and mortality (proportion of painters in the population)? What is the public health impact of painting on respiratory health compared to other occupational exposures (mining, construction,….)? What about different types of paints (solvent-based vs water-based paints) and application modes (spraying vs brush)?**

**Response:** We appreciate your valuable suggestions. We have incorporated the following sentences to address the gaps and have made the appropriate revisions in the discussion.

“It can be inferred from the current review that paint industry workers exposed to VOCs exhibit greater respiratory health impacts such as a decline in pulmonary function. Furthermore, this review served as a needs assessment, allowing us to identify gaps in the current knowledge of pulmonary function among painters. Limited research assessed the pulmonary function in developed countries and respiratory symptoms in Asian countries. There is a dearth of sufficient evidence about the influence of VOCs on painters working in unorganized sectors such as construction and paint manufacturing, as compared to organized sectors such as the automobile industry. The prevalence of respiratory ailments, including COPD and pulmonary fibrosis, among painters is not well-documented. However, other meta-analyses have reported that the pooled risk ratio of studies that evaluated the impact of VOC exposure on asthma was higher, particularly for exposures to benzene, toluene, and p-dichlorobenzene (50). A detailed assessment is needed to assess the impacts of solvent-based paints against water-based paints, as well as the differences between spray painting and brushing/rolling methods. Therefore, it is crucial to do further study in this topic to ascertain the actual impact.”

**Page: 23,24	Lines:398 -407.**

**Query #15: Language: generally OK, but some editing is needed (pulmonary function is generally singular; "the effect reduced", ...).**

**Response:** We have incorporated the reviewer’s suggestions and made the necessary changes in the entire manuscript.

**Journal Requirements:**

**1. Please review your reference list to ensure that it is complete and correct. If you have cited papers that have been retracted, please include the rationale for doing so in the manuscript text, or remove these references and replace them with relevant current references. Any changes to the reference list should be mentioned in the rebuttal letter that accompanies your revised manuscript. If you need to cite a retracted article, indicate the article’s retracted status in the References list and also include a citation and full reference for the retraction notice.**

After a thorough review of the entire reference list, we did not find any articles that have been retracted. However, some of the listed references were not cited properly in the bibliography, so we have made the necessary changes. Furthermore, kindly inform us if any references require correction or revision. Your feedback is greatly appreciated and we will make the necessary adjustments accordingly.

**Ref No 32** Aribo.E.O, Antai A.B. Lung function parameters in spray painters in Calabar,Nigeria.Ann Biol.Res. 2014 Dec ; 5(11):32-5.

**Ref No.42** Mukhtar M, Gotmare P. Health hazard and occupational safety challenges for Unorganized sector workers in India. J Contemp Issues Bus Gov. 2021 Jun Feb 9;27 (1):3210-21.

**2. While revising your submission, please upload your figure files to the Preflight Analysis and Conversion Engine (PACE) digital diagnostic tool, https://pacev2.apexcovantage.com/. PACE helps ensure that figures meet PLOS requirements.**

As per your suggestion we have upload our figures files to the Preflight Analysis and Conversion Engine (PACE) digital diagnostic tool and converted the figures to meet PLOS requirements.
