# Supplementary material for: Respiratory symptoms and pulmonary function in paint industry workers exposed to volatile organic compounds: A systematic review and meta-analysis
Source: PLoS One. 2024 Dec 26;19(12):e0315464. doi: 10.1371/journal.pone.0315464 (PMC11671006; doi:10.1371/journal.pone.0315464)
Supplement: Attachment — Submitted filename: Response to reviewer.docx [file pone.0315464.s006.docx]

**PONE-D-24-08732**

**Respiratory symptoms and Pulmonary function in paint industry workers: A Systematic Review and Meta-Analysis**

**Dear Editor in-chief,**

We sincerely appreciate your consideration of our work and the thorough peer review process that has helped refine our manuscript to meet PLOS ONE’s standards.

**Reviewer #1: The authors have satisfactorily addressed my comments/questions.** **I have only few editorial issues to mention:**

We appreciate your scholarly peer review process and the time you have spent to share your thoughtful suggestions with us. Your input is greatly appreciated.

**#Comment 1**

**Title: consider specifying in the title that the review focuses on exposure to VOLATILE ORGANIC COMPOUNDS in painters**

Thank you for your valuable comment. As per your suggestions, we have specified VOC exposure in the title

**New Title:** *Respiratory symptoms and Pulmonary function in paint industry workers exposed to Volatile Organic Compounds: A Systematic Review and Meta-Analysis*

**#Comment 2: line 179: "America", specify North America or United States of America**

Thank you for your valuable suggestions. We have specified America as United States of America

**#Comment 3:** **lines 382-3: clarify sentence**

As per your suggestion we have rephrased the sentence for better clarity

**Rephrased sentence:** Although publication bias existed studies that assessed the pulmonary function parameters, Fail-safe (sensitivity) analysis showed that a substantially greater number of studies with null values would be required to negate the present findings.

**#Comment 4: line 385: the present review HAS certain limitations**

Thank you for identifying the typo error

**#Comment 5:** **line 388: I suggest replacing "increase" and "decrease ..." by "excess" and "lower mean pulmonary function" compared to controls ("increase" and "decrease" suggest a temporal process that cross-sectional studies cannot assess)**

Following your suggestion, we have changed the two terms.

**#Comment 6:** **line 398: "greater respiratory impact": greater than what or who ? I suggest to replace this by "adverse respiratory health impacts"**

As per your suggestion we have replaced the words

**#Comment 7:** **line 413: see comment above: replace "notable increase" and "decline" by "higher prevalence" and "lower average", respectively**

Following your suggestion, we have changed the two terms.

**#Comment 8:** **references: some references mention first names in full: 16, 22, 26, 38; reference 44 has strange author names.**

Thank you for pointing out the errors. We have made the necessary changes according to your suggestion.
